# Ophthalmoplegia and Slurred Speech in an Intravenous Drug User

**DOI:** 10.1371/journal.pmed.0030453

**Published:** 2006-12-26

**Authors:** Jaime Vera, Anke Hensiek, Charles Woodrow, Francesca Crawley, Sanjeev Krishna

## Abstract

A 35-year-old heroin user presented with acute, progressive diplopia and slurred speech. Krishna and colleagues discuss the diagnosis and management of this patient.

## DESCRIPTION of CASE

A 35-year-old unemployed man presented to Accident and Emergency with a three-day history of progressive diplopia and a one-day history of slurred speech. There was no history of fever, paraesthesiae, dysphagia, or respiratory difficulty. He admitted drug abuse including “muscling” (intramuscular injection of heroin).

On examination, the patient was well, ambulant, alert, and orientated. Vital signs were normal and oxygen saturation was 97% (room air). There were injection sites in both arms but no infected wounds. There were no cardiac murmurs. The rest of the general examination was normal.

Visual fields were full and fundi normal. Bilateral partial ptosis and complete bilateral ophthalmoplegia were obvious. Pupils were dilated and poorly responsive to light, without an afferent pupillary defect. Facial muscle power was normal. There was dysarthria with absent palatal movements but a normal gag reflex. No other abnormal neurology was present; limb power was normal with preserved deep tendon reflexes and no ataxia. The gait was normal.

Full blood count, and renal and liver function tests were within normal limits. C-reactive protein was 3 mg/l (normal <10 mg/l). Blood cultures and serum were sent for microbiological testing. Chest X ray and electrocardiogram were normal. The patient was admitted for observation.

### What Is the Likely Diagnosis?

Over a period of three days this patient has developed symmetrical paralysis of the ocular and oropharyngeal muscles without long-tract signs or sensory deficit. Is the disease central or peripheral in origin? A central cause is difficult to envisage since it would involve several cranial nerve nuclei yet spare other structures such as corticospinal tracts. Peripheral causes may act at the level of motor nerves, neuromuscular junction, or muscle. A primary muscular cause is unlikely because of the rapid onset of symptoms. Motor neuropathies may be acute, e.g., a cranial variant of Guillain-Barré syndrome; however features such as antecedent illness, paraesthesiae, and loss of reflexes would be expected.

Myasthenia gravis is a common neuromuscular disorder that may present relatively acutely. However, pupillary dilatation is not observed because antibodies that mediate this disease are specific for nicotinic acetylcholine receptors, thereby sparing the parasympathetic nervous system. Furthermore, total ophthalmoplegia would be very unusual.

The clinical syndrome is typical of wound botulism. Botulinum toxin acts pre-synaptically at all peripheral cholinergic synapses, blocking neuromuscular junctions as well as parasympathetic and sympathetic pathways. Cranial nerves are affected before descending weakness of voluntary muscles develops. Typical early symptoms are therefore dry mouth, blurred vision, diplopia, dysphagia, dysphonia, and dysarthria. Pupillary dilatation is common. Limb weakness and respiratory compromise may follow rapidly, and the latter may occur without the former. Reflexes are present unless flaccid paralysis becomes severe enough to suppress them. The toxin does not cross the blood–brain barrier. Sensory disturbance is not a feature of botulism.

A diagnosis of wound botulism is also suggested by the history of intramuscular drug injection. The incidence of wound botulism following heroin injection into skin or muscle has risen dramatically in the United Kingdom since 2000 [1]. The site of infection may appear benign (as in this case) [2], and fever is absent unless wounds are co-infected. Gastrointestinal symptoms are not seen in wound botulism.

Although unlikely, other differential diagnoses to consider include diphtheria, tick paralysis, carbon monoxide, or organophosphate poisoning.

### What Measures Should Be Instituted Without Delay?

Wound botulism now carries a case fatality rate of 10%–15% compared to rates of 60%–70% in the early 20th century. This reduction follows the development of intensive care; most patients with botulism require assisted ventilation during the course of their illness and the cornerstone of early management is prompt recognition and optimal treatment of neuromuscular respiratory failure. The patient should be placed in a high-intensity facility with regular monitoring of vital capacity; a vital capacity less than 30% of predicted indicates impending respiratory failure. Patients should not eat or drink because of the risk of aspiration; nutritional support (enteric or parenteral) should be instituted.

### Progress

The patient was transferred to a neurological ward for monitoring and remained nil by mouth. Twelve hours after admission he developed dysphagia and weak neck flexion and extension as well as loss of gag reflex. Vital capacity was 2.0 l (39% of predicted) and arterial blood gas (room air) showed pH 7.41, pCO_2_ 4.1 kPa (normal 4.5–6), and pO_2_ 10 kPa (normal 11–14).

Twenty hours after admission the patient became breathless and vital capacity was 0.98 l with arterial blood gas (100% oxygen) showing pH 7.29, pCO_2_ 5.8 kPa, and pO_2_ 11.9 kPa. The patient was electively intubated and ventilated.

### What Is the Specific Treatment?

Given the inherent delay in microbiological confirmation of botulism, botulinum antitoxin should be administered as soon as possible after clinical diagnosis. Botulinum antitoxin administration is associated with lower mortality and shortened clinical course in wound botulism [3]. Antitoxin binds free, not intraneuronal, neurotoxin; early administration does not reverse the course of intoxication but prevents worsening paralysis. Early administration of antitoxin is associated with improved outcomes [4,5].

A single dose of trivalent (against types A, B, and E) or heptavalent (against all types) antitoxin neutralises all free neurotoxin, and its half-life of five to eight days indicates that further doses are not helpful if the infection itself is treated (see below). Antitoxin is obtained from a reference centre (in the UK, the Health Protection Agency, telephone 0208-200-6868). 10–15 ml serum should be taken prior to administration of antitoxin (which rapidly neutralises circulating toxin and renders diagnosis impossible).

Antitoxin is an equine derivative and may cause anaphylactic reactions; skin testing for sensitivity prior to administration is suggested. If there is no reaction, antitoxin is administered intravenously, with resuscitation facilities to hand.

In wound botulism the toxin is produced in vivo (in food botulism toxin is preformed). Treatment of infected wounds is required to eradicate the source of toxin. Benzylpenicillin and metronidazole are appropriate antibiotics; gentamicin may exacerbate neuromuscular blockade. Thorough surgical debridement is vital (ideally after antitoxin administration so that released toxin is neutralised).

The management of botulism in relatively resource-poor settings is difficult given the importance of intensive care; antitoxin can be obtained from overseas [6], and organism eradication applied wherever a case presents.

### Treatment

Fifteen hours following admission, the patient received a single dose of trivalent botulinum antitoxin one hour after a negative skin test (0.1 ml intradermal antitoxin). A course of intravenous benzylpenicillin was administered.

### How Is the Diagnosis Confirmed?

Definitive diagnosis of wound botulism can only be performed in a reference laboratory and requires culture of Clostridium botulinum on anaerobic media from tissue/pus or identification of botulinum toxin in serum or other samples. The earliest available serum sample should be tested. Specific serum toxin detection is conventionally by a mouse lethality assay which has a sensitivity of 50% or less [1,2]. Immunoassays detecting toxin from a variety of clinical sources match the sensitivity of bioassay and provide results relatively quickly, but are not yet optimised to detect all neurotoxin subtypes.

Overall, of UK wound botulism cases reported since 2000, 42% were confirmed by toxin detection or culture [1]. Hence diagnosis often rests on clinical assessment supported by neurophysiological studies (see below); brain MRI, cerebrospinal fluid examination and measurement of anti-acetylcholine receptor antibodies should be undertaken to exclude differential diagnoses such as brain-stem infarct, Guillain-Barré syndrome, and myasthenia gravis.

### Results of Investigations

Botulinum antitoxin bioassay on admission serum was negative. Nerve conduction studies showed no evidence of demyelinating neuropathy and normal sensory responses. Electromyography demonstrated polyphasic motor unit potentials, with small amplitude motor responses more than doubling in size after exercise. There were decrements on repetitive stimulation at low stimulus frequencies with an increment at higher rates of stimulation. These findings indicate reversible pre-synaptic neuromuscular junction blockade, and are typical of botulism [7]. MRI brain was normal, examination of cerebrospinal fluid revealed no evidence of inflammation or elevation in protein level, and anti-acetylcholine receptor and HIV antibodies were negative.

The clinical picture of descending paralysis (in a patient practising intramuscular heroin injection) accompanied by electrophysiological features of botulism, with normal ancillary investigations, provides strong evidence for a diagnosis of wound botulism.

### Progress

Neurological function began to improve (see Video S1 performed in intensive care) and extubation was performed after 72 hours. Seven days after admission all bulbar signs had resolved and oral intake was resumed. The patient was discharged three days later and when reviewed in the out-patient department at one month had no neurological problems.

## DISCUSSION

Wound botulism occurs when spores of C. botulinum contaminate a wound, germinate, and produce botulinum neurotoxin in vivo. Botulinum toxin cleaves synaptosome-associated proteins that mediate fusion of vesicles with the pre-synaptic membrane in cholinergic synapses, thereby inhibiting release of acetylcholine. The cellular receptors for both A and B toxin types have now been identified [8].

Infections by a variety of spore-forming bacteria, particularly anaerobic organisms producing neurotoxins (C. botulinum and tetani) and histotoxins (C. novyi and others), have undergone a dramatic increase in incidence in the UK in the last five years [9]. The reasons for this remain unclear but may involve contamination of specific batches of heroin as well as changes in injection practices. Since 2000, more than 120 cases of wound botulism have been reported in the UK, where this is now the most common aetiology of botulism [1].

**Key Learning Points**

- In heroin users who inject into skin or muscle, think of spore-forming bacterial diseases (botulism, tetanus as well as soft tissue infection with severe illness).
- Wound botulism should always be considered in patients with ophthalmoplegia.
- Common early symptoms include diplopia and blurred vision although respiratory compromise may occur before oculobulbar weakness.
- Other typical features are: descending symmetrical progression; absence of altered mental status or sensory abnormality; infected wounds may not be clinically apparent.

**Treatment of wound botulism**

- Early detection and management of neuromuscular respiratory failure; intense observation for markers of impending respiratory failure is vital.
- Early administration of antitoxin (when clinical diagnosis made).
- Antibiotics (avoid gentamicin).
- Wound debridement when necessary.

## Supporting Information

Video S1 Neurological Examination Demonstrating Physical Signs of Botulism After testing for pupillary reaction to light, the patient is asked to look to the left, right, up, and down. Signs include bilateral ptosis, loss of pupillary reaction to light, and bilateral ophthalmoplegia (with loss of oculocephalic reflex). Power and deep tendon reflexes in the limbs are preserved.
(5.6 MB MPG)

## References

[pmed-0030453-b001] Health Protection Agency (2006). Wound botulism in injecting drug users in the United Kingdom.

[pmed-0030453-b002] Brett MM, Hallas G, Mpamugo O (2004). Wound botulism in the UK and Ireland. J Med Microbiol.

[pmed-0030453-b003] Tacket CO, Shandera WX, Mann JM, Hargrett NT, Blake PA (1984). Equine antitoxin use and other factors that predict outcome in type A foodborne botulism. Am J Med.

[pmed-0030453-b004] Sandrock CE, Murin S (2001). Clinical predictors of respiratory failure and long-term outcome in black tar heroin-associated wound botulism. Chest.

[pmed-0030453-b005] Chang GY, Ganguly G (2003). Early antitoxin treatment in wound botulism results in better outcome. Eur Neurol.

[pmed-0030453-b006] Reller ME, Douce RW, Maslanka SE, Torres DS, Manock SR (2006). Wound botulism acquired in the Amazonian rain forest of Ecuador. Am J Trop Med Hyg.

[pmed-0030453-b007] Cherington M (1982). Electrophysiologic methods as an aid in diagnosis of botulism: A review. Muscle Nerve.

[pmed-0030453-b008] Dong M, Yeh F, Tepp WH, Dean C, Johnson EA (2006). SV2 is the protein receptor for botulinum neurotoxin A. Science.

[pmed-0030453-b009] Brett MM, Hood J, Brazier JS, Duerden BI, Hahne SJ (2005). Soft tissue infections caused by spore-forming bacteria in injecting drug users in the United Kingdom. Epidemiol Infect.

